# Polymorphism of rs599839 in the *PSRC1* gene is associated with coronary artery disease in an Iranian population

**DOI:** 10.34172/jcvtr.2023.31742

**Published:** 2023-09-23

**Authors:** Golnaz Houshmand, Mohammad Javad Alemzadeh-Ansari, Saeideh Mazloumzadeh, Niloofar Naderi, Maryam Pourirahim, Katayoun Heshmatzad, Majid Maleki, Samira Kalayinia

**Affiliations:** ^1^Rajaie Cardiovascular Medical and Research Center, Iran University of Medical Sciences, Tehran, Iran; ^2^Cardiovascular Intervention Research Center, Rajaie Cardiovascular Medical and Research Center, Iran University of Medical Sciences, Tehran, Iran; ^3^Cardiogenetic Research Center, Rajaie Cardiovascular Medical and Research Center, Iran University of Medical Sciences, Tehran, Iran

**Keywords:** Coronary artery disease, *PSRC1*, Case-control study, Polymorphism, Risk factor

## Abstract

**Introduction:**

Coronary artery disease (CAD) is the leading health complication worldwide because of its high prevalence and mortality. The association between CAD susceptibility and the rs599839 (C/T) polymorphism in the human proline and serine-rich coiled-coil (*PSRC1*) was reported in a genome-wide association study. To validate this association, we performed this case-control study to genotype the 1p13.3 (rs599839) locus in a sample of the Iranian population with CAD (stenosis≥70% in≥1 coronary artery).

**Methods:**

We performed an association analysis with PCR and Sanger sequencing of rs599839 (C/T) polymorphism and CAD risk in 280 CAD patients and 287 healthy controls defined as a coronary calcium score of zero and no noncalcified plaques in coronary computed tomography angiography. SPSS, version 16.0, was applied for statistical analysis.

**Results:**

The rs599839 (C/T) locus showed a significant association with CAD (*P* value<0.001). TT and CT genotypes were associated with CAD (*P* value<0.001). Furthermore, the dominant status (TT+CT vs. CC) was associated with an increased risk of CAD (OR, 9.14; 95% CI, 3.77 to 22.15; and *P* value<0.001).

**Conclusion:**

The study findings indicate strong evidence for rs599839 (C/T) association with CAD risk.

## Introduction

 Coronary artery disease (CAD) is the principal cause of death the world over. Although environmental factors, including hypertension, hypercholesterolemia, smoking, and diabetes mellitus, affect the development of CAD,^1^ genetic factors constitute one of the major agents in its pathogenesis, accounting for about 50% of patients.^2^ In the last decade, genome-wide association studies (GWASs) have determined some potential loci for CAD.^3-5^ Nonetheless, the reported associations require validation by sizable studies on the role of CAD-related loci.^6^ The proline and serine-rich coiled-coil (*PSRC1*) gene participates in processes that change plasmatic cholesterol and blood lipid levels, increasing susceptibility to cardiovascular disease.

 The Wellcome Trust Case Control Consortium (WTCCC) study on 1926 patients and 2938 controls reported strong associations between the chromosome 1p13.3 (rs599839) locus in *PSRC1 *(NM_032636.7) and CAD (OR for the common risk allele, rs599839 (T), 1.29; 95% CI, 1.18 to 1.40; and adjusted *P *value = 0.0006).^7^ Another study performed an association analysis of rs599839 and CAD risk on 11550 patients and 11205 controls from 9 European studies and confirmed this polymorphism association (OR, 1.13; 95% CI, 1.08 to 1.19; and P value = 1.44 × 10−7).^8^

 We conducted the present study to evaluate the association between the rs599839 (C/T) polymorphism and CAD in a sample of the Iranian population.

## Materials and Methods

###  Study Population, Consent to Participate, and Ethics Approval 

 The population of the present case-control study consisted of 567 unrelated Iranian individuals: 280 CAD patients (214 men and 66 women) and 287 controls (162 men and 125 women) referred to Rajaie Cardiovascular Medical and Research Center, Iran University of Medical Sciences, Tehran, Iran, between June 2020 and December 2021, because either they had exhibited symptoms or they needed a regular medical checkup.

 All the study participants were younger than 65 years and had laboratory tests, including triglyceride, high-density lipoprotein, total cholesterol, low-density lipoprotein, fasting blood sugar, hemoglobin A1C, blood urea nitrogen, creatinine, and hemoglobin; echocardiography (left ventricular ejection fraction); angiography; computed tomography angiography; and physical examinations, including systolic and diastolic blood pressure and heart rate.

 In the present study, CAD was defined as stenosis ≥ 70% in one or more coronary arteries; risk factors, including a family history of CAD, smoking, hypertension (systolic blood pressure ≥ 130 mm Hg or diastolic blood pressure ≥ 90 mm Hg), dyslipidemia (total cholesterol ≥ 6.22 mmol/l), diabetes mellitus (fasting blood sugar ≥ 11.1 mmol/l or hemoglobin A1c ≥ 6.5%), and addiction; symptoms, including typical or atypical chest pain and dyspnea; prior percutaneous coronary interventions; prior coronary artery bypass grafting; stent placement; chronic kidney disease; and chronic obstructive pulmonary disease.

 The control group was composed of individuals with no history of cerebrovascular, peripheral, and congenital heart disease and no atherosclerotic plaques, defined as an Agatston coronary calcium score of zero, and no noncalcified plaques in coronary computed tomography angiography with a dual-source computed tomography scanner (the SOMATOM Force).

 A questionnaire containing information about the participants was completed. Blood samples were taken from the entire study population in compliance with the Declaration of Helsinki. Ethical approval was obtained from the Ethics Committees of Rajaie Cardiovascular Medical and Research Center, Iran University of Medical Sciences, Tehran, Iran (IR.RHC.REC.1399.021). Informed consent was obtained for study participation.

###  Sampling, DNA Extraction, and Allelic Differentiation

 Fasting peripheral blood samples were collected for biochemical, lipid, platelet, and homocyte evaluation. DNA was extracted from 2 mL of EDTA blood samples via the salting-out method with the DNSol Midi kit (Roche: Product No. 50072012). White blood cells were separated after the lysis of red blood cells and subjected to lysis with special ionic detergents. Contaminants, such as protein, were precipitated via the salting-out method. DNA was precipitated using absolute ethanol or isopropanol before being washed with 70% ethanol to remove salts and perform rehydration with a freshly made rehydration buffer. The quality and quantity of the extracted DNA were checked via agarose gel electrophoresis on a NanoDrop (Thermo Fisher Scientific, U.S.A.).

 The c.1002 + 10C > T in *PSRC1* (rs599839) was selected for the current study and evaluated using polymerase chain reaction (PCR) and Sanger sequencing.

**Figure 1:**
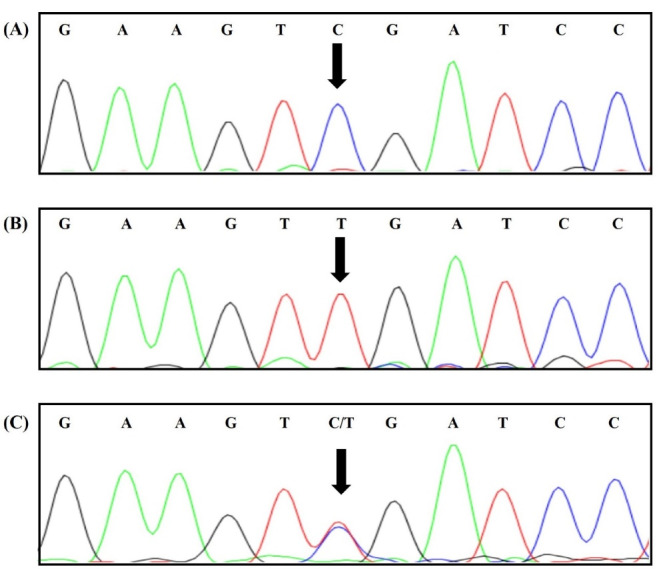
The partial chromatograms indicate CC **(A)**, TT **(B)**, and CT **(C)** genotypes

 Primers were designed with Primer3, version 4.1.0 (https://primer3.ut.ee/): forward 5-TGGGGTGGATTAGGATTGAGC-3′ and reverse 5′-GCCTGTCCTTGTCTAATGCCA-3′. PCR was performed on a SimpliAmp Thermal Cycler (Thermo Fisher Scientific) with 100 ng of DNA, 1.5 mmol/L of MgCl2, 200 mmol/L of dNTP, 10 pmol/L of the primers, and 1 U of Taq DNA polymerase (Amplicon, U.K.). Incubation was performed at 95 °C for 5 minutes and 35 amplification cycles (30 seconds at 95 °C, 30 seconds at 62 °C, and 30 seconds at 72 °C). The PCR products were analyzed via agarose gel (2%) electrophoresis and sequenced on an ABI Sequencer 3500XL PE (Applied Biosystems) using the same primer sets. The sequences were analyzed with CodonCode Aligner, version 7.1.2 (Figure 1).

###  Statistical Analysis 

 The Kolmogorov-Smirnov test was used to evaluate the distribution of variables. Values were expressed as mean ± standard deviation and numbers (percentages), as appropriate. Comparisons were performed using the independent* t*-test or ANOVA for normally distributed variables, the Mann-Whitney or Kruskal-Wallis test for non-normally distributed variables, and the chi-square or Fisher exact test for categorical variables. Logistic regression models were applied to determine the association between the rs599839 (C/T) polymorphism and CAD.

*P *values < 0.05 were considered significant. All the analyses were performed using SPSS, version 16.0 (SPSS Inc., Chicago, IL., U.S.A.).

## Results

###  Characteristics of the CAD and control groups

 We analyzed 280 CAD patients (mean age = 55 years) and 287 controls (mean age = 39 years). Table 1 compares clinical characteristics between the CAD and control groups. The proportions of male gender, smoking, dyslipidemia, hypertension, and diabetes were higher in the CAD group than in the control group (all *P *values < 0.05). The mean values of age, height, systolic and diastolic blood pressure, heart rate, and left ventricular ejection fraction differed between the two groups (all *P *values < 0.05).

**Table 1:** Characteristics of the CAD and control groups

**Characteristic**	**CAD (n=280)**	**Control (n=287)**	***P***** value**
Age	55.43 ± 6.59	39.42 ± 6.99	**<0.001**
Male, n (%)	214 (76.4)	162 (56.4)	**<0.001**
Height (cm)	168.30 ± 9.44	171.25 ± 9.70	**<0.001**
Weight (kg)	80.45 ± 13.60	82.07 ± 15.49	0.196
BMI (kg/h2)	28.43 ± 4.44	27.91 ± 4.33	0.168
Smoking, n (%)	113 (40.5)	45 (15.9)	**<0.001**
FH of CAD	127 (45.7)	131 (46.3)	0.885
Dyslipidemia	131 (47.1)	41 (14.5)	**<0.001**
Hypertension, n (%)	166 (59.5)	55 (19.4)	**<0.001**
Diabetes, n (%)	107 (38.4)	18 (6.4)	**<0.001**
Addiction	19 (7.0)	5 (1.8)	**0.002**
Typical CP, n (%)	231 (82.8)	85 (30.0)	**<0.001**
Atypical CP, n (%)	84 (30.8)	101 (35.7)	0.218
Dyspnea, n (%)	123 (44.4)	114 (40.3)	0.324
Prior PCI, n (%)	96 (34.4)	0 (0.0)	**<0.001**
Prior CABG, n (%)	24 (8.6)	0 (0.0)	**<0.001**
CKD, n (%)	2 (0.7)	1 (0.4)	0.621
COPD, n (%)	2 (0.7)	1 (0.4)	0.621
SBP, mm Hg	126.22 ± 17.54	125.25 ± 15.57	0.495
DBP, mm Hg	78.63 ± 10.15	80.65 ± 13.04	**0.042**
HR	76.91 ± 7.09	79.96 ± 9.28	**<0.001**
LVEF, (%)	46.65 ± 9.09	54.86 ± 5.24	**<0.001**

CAD, coronary artery disease; FH, family history; CP, chest pain; PCI, percutaneous coronary intervention; CABG, coronary artery bypass grafting; CKD, chronic kidney disease; COPD, chronic obstructive pulmonary disease; SBP, systolic blood pressure; DBP, diastolic blood pressure; HR, heart rate; LVEF, left ventricular ejection fraction Compared with the control group: the numbers shown in bold are statistically significant at *P* values < 0.05

###  Distribution of the rs599839 (C/T) polymorphism genotypes of the PSRC1 gene in the CAD and control groups

 The rs599839 (C/T) polymorphism of *PSRC1* was genotyped in the CAD and control groups. Table 2 displays the distribution of genotypes in the two groups. The proportions of CT and TT genotypes were higher in the CAD group than in the control group (*P *value < 0.001). The proportions of both dominant (TT + CT vs. CC) and recessive (CC + CT vs. TT) rs599839 polymorphisms were higher in the case group (*P* value < 0.001 for both) (Table 2). The proportions of participants with the T allele in the CAD and control groups were 84.8% and 37.6%, respectively, and those of participants with the C allele were 15.2% and 62.4%, respectively (*P *value < 0.001). The association between the rs599839 (C/T) of *PSRC1* and CAD risk factors was evaluated. Table 3 reveals that the proportions of the TT genotype in individuals with smoking, hypertension, diabetes, and dyslipidemia were 66.5%, 64.7%, 69.6%, and 68.6%, respectively.

**Table 2:** Genotype and allele frequencies in the CAD and control groups

**Polymorphism**	**Status**		**CAD (n=280), n (%)**	**Control (n=287), n (%)**	***P***** value**	**OR (95% CI)**
rs599839	Genotypes	CC	22 (7.9)	165 (57.5)	**<0.001**	1.00
		CT	41 (14.6)	28 (9.8)		10.98 (5.74 to 21.14)
		TT	217 (77.5)	94 (32.8)		17.31 (10.43 to 28.73)
	Recessive	TT	217 (77.5)	94 (32.8)	**<0.001**	1.00
		CC + CT	63 (22.5)	206 (67.2)		5.86 (9.68 to 26.00)
	Dominant	CC	22 (7.9)	165 (57.5)	**<0.001**	1.00
		TT + CT	258 (92.1)	122 (42.5)		7.07 (4.87 to 10.27)

CAD, coronary artery disease The numbers shown in bold are statistically significant at *P* values < 0.05 for the genotype and allele frequencies of the polymorphism

**Table 3:** The CAD-associated risk factors among rs599839 different genotypes

**Parameter**	**CC genotype (n=187)**	**CT genotype (n=69)**	**TT genotype (n=311)**	**t/x**^2^	***P***** value**
Smoking	32 (20.3)	21 (13.3)	105 (66.5)	15.914	**<0.001**
Hypertension, n (%)	48 (21.7)	30 (13.6)	143 (64.7)	20.274	**<0.001**
Diabetes, n (%)	19 (15.2)	19 (15.2)	87 (69.6)	22.472	**<0.001**
Dyslipidemia	29 (16.9)	25 (14.5)	118 (68.6)	28.703	**<0.001**

CAD, coronary artery disease; FH, family history The numbers shown in bold are statistically significant at *P* values < 0.05

###  CAD risk factors

 To determine whether the rs599839 polymorphisms of the *PSRC1* gene were independent parameters for CAD, we adjusted confounding risk factors, such as age, sex, body mass index, smoking, dyslipidemia, diabetes, and hypertension (Table 4). The dominant model (TT + CT vs. CC) of rs599839 presented a remarkable independent association with CAD (OR, 9.14; 95% CI, 3.77 to 22.15; and *P *value < 0.001).

**Table 4:** The regression analysis of the CAD and control groups

**Risk factor**	**OR**	**95% CI**	**Wald**	***P***** value**
rs599839 (TT + CT vs. CC)	9.14	3.77 to 22.15	23.993	**<0.001**
Age	1.33	1.25 to 1.42	73.351	**<0.001**
Sex (male)	3.98	1.51 to 10.49	7.785	**0.005**
BMI	1.07	0.97 to 1.17	1.830	0.176
Smoking	2.62	1.05 to 6.53	4.266	**0.039**
Dyslipidemia	1.84	0.76 to 4.45	1.846	0.174
Diabetes, n (%)	11.47	3.42 to 38.42	15.646	**<0.001**
Hypertension, n (%)	2.27	1.01 to 5.11	3.956	**0.047**

Adjusted for age, sex, BMI, smoking, dyslipidemia, diabetes, and hypertension. CAD, coronary artery disease; OR, odds ratio; CI, confidence interval The numbers shown in bold are statistically significant at *P* values < 0.05

## Discussion

 To the best of our knowledge, our study is the first investigation of rs599839 C > T in the Iranian population, while the literature features a few studies on the association between this polymorphism and CAD around the world. We also defined our healthy population meticulously by using coronary computed tomography angiography to select individuals with no coronary atherosclerotic plaques, a criterion not used in similar studies.

 In modern cardiology, the use of high-end coronary computed tomography angiography assists cardiologists in detecting atherosclerotic plaques earlier and modifying further management for prognostic benefits.

 The association detected in the present study was also indicated in 10 other studies, namely those by including Samani et al,^8^ Samani et al,^9^ Ronald et al,^10^ Xu et al,^11^ Zhou et al^12^ Rodríguez-Arellano et al^13^ Angelakopoulou et al^14^ Ellis et al,^15^ Lee et al,^16^ and Yan et al.^17^ For the first time, in 2008, Huang et al^18^ reported an association between the rs599839 polymorphism and low-density lipoprotein and premature CAD in 615 Chinese Han individuals. Recently, Rodríguez-Arellano et al^13^ showed that rs599839 was significantly associated with CAD (additive OR, 0.72 and *P* value = 0.009; dominant OR, 0.66 and *P *value = 0.007) in a sample of 907 Mexican individuals (394 CAD cases and 513 controls). Many studies have identified an association between rs599839 (T) and higher low-density lipoprotein levels.^19-22^ Wallace et al^19^ reported that the T allele was associated with a 6% increase in non-fasting serum low-density lipoprotein in over 4000 Caucasian Europeans. Therefore, the CAD-associated locus of the *PSRC1* gene probably increases CAD risk by impacting the low-density lipoprotein level. A GWAS of the Framingham Heart Study (FHS) data indicated an association between rs599839 (T) and serum total cholesterol and high-density lipoprotein levels.^23^ Prior investigations have also determined an association between rs599839 and triglyceride metabolism.^21,24^ Such evidence supports the role of the rs599839 polymorphism in cholesterol metabolism and CAD risk.

 Our results also demonstrated associations between rs599839 and smoking, hypertension, diabetes, and dyslipidemia, which might denote the impact of rs599839 on CAD. Qin J et al^25^ concluded that the G allele of rs599839 was a protective factor against diabetic peripheral artery disease. The same result concerning the protective effect of rs599839 (G) was observed against dyslipidemia and cardiovascular diseases.^26^ Several studies have stated that the sequence variant on 1p13.3, a rare G allele of rs599839, is associated with aortic aneurysm independently.^27,28^ Nevertheless, CAD risk may result only from the T allele of the rs599839 polymorphism, and the G allele may have a protective impact.

 The results of the current study should be interpreted in light of its limitations. Firstly, the study population was small, and a large cohort should be surveyed to identify the association between the rs599839 polymorphism and CAD. However, we selected the case and control groups thoroughly. Indeed, our control group was selected from individuals with no atherosclerotic plaques in coronary computed tomography angiography. Secondly, we lacked information regarding the levels of risk factors in most of the control group.

 Further clinical and basic research is required to identify the specific effects of rs599839 on CAD and the mechanisms involved.

## Conclusion

 The results of the present case-control study showed that the T allele of the rs599839 polymorphism was associated with CAD. An association was also found between rs599839 and diabetes and hypertension. Additionally, the results indicated a significant association between the *PSRC1* gene and CAD. Therefore, the rs599839 polymorphism in the *PSRC1* gene might be a suitable target for CAD treatment and its risk factor alleviation.

## Acknowledgments

 The authors wish to acknowledge the kind collaboration of Mrs. Pantea Vaght Mobaraki for blood sampling. This research was funded by the Cardiogenetics Research Center, Rajaie Cardiovascular Medical and Research Center, Tehran, Iran.

## Competing Interests

 The authors declare that they have no competing interests.

## Ethical Approval

 The study protocol was approved by the Ethics Committee of Rajaie Cardiovascular Medical and Research Center, Iran University of Medical Sciences, Tehran, Iran (IR.RHC.REC.1399.021). Informed consent was obtained for study participation.

## Funding

 The authors received no specific funding for this research.
